# Domestication of proteins – from evolution to revolution

**DOI:** 10.1111/1751-7915.13987

**Published:** 2021-12-01

**Authors:** John van der Oost

**Affiliations:** ^1^ Laboratory of Microbiology Wageningen University & Research Wageningen Netherlands

In the course of natural evolution, an overwhelming diversity of proteins has emerged. Collectively, these proteins are responsible for a wide range of biological functions that, in one way or another, support live. Billions of years of natural selection have resulted in survival of the fittest protein variants that function appropriately in the context of a biological entity. When using these proteins for biotechnology applications, however, it is often required to improve their performance, because of distinct conditions (*in vitro*, *ex vivo*, *in vivo*) and different demands (activity, specificity, stability).

Hence, repurposing natural proteins for biotechnological applications generally requires domestication, aiming at optimising their functionality by adjusting their amino acid sequence. Rational engineering approaches aim at specifically substituting one or more amino acid residues by engineering of the corresponding gene. Rational design obviously requires a relatively high level of understanding of structural and functional features of the protein of interest. In case insights are lacking on how to rationally improve a certain protein's functionality, laboratory evolution is an attractive alternative. The impact of laboratory evolution in optimising proteins is reflected by the Nobel Prize in Chemistry 2018, awarded to Frances H. Arnold, George P. Smith, and Gregory P. Winter.

Like natural evolution, laboratory evolution is based on repeated cycles of genetic variation, expression and selection (Stemmer, 1994; Arnold, 2018). To allow tracing a protein variant with a desired functionality back to its gene, a genotype‐to‐phenotype linkage is a key requirement. This can be achieved either by physically linking the gene and gene‐encoded product (DNA display, mRNA display, ribosome display), or by compartmentalising the gene and the corresponding protein within the same physical space (reviewed by Bouzetos et al., 2021). Unicellular microorganisms (e.g. *E*. *coli*) or viral particles (e.g. M13) are often used as biological micro‐compartments.

Despite spectacular technical and biochemical progress, laboratory evolution systems are often technically challenging. Successful applications rely on efficient genetic variation, robust protein production, and smart screening/selection of improved variants. In addition, especially in case of huge libraries (a million variants or more), the process can be rather laborious and/or expensive. A spectacular development concerns a Phage‐Assisted Continuous Evolution system (Esvelt et al., 2011). In this PACE approach, M13 phages carry a gene encoding a protein‐of‐interest that controls the production of functional phage particles in a mutator *E*. *coli* host. The fitness of released M13 particles directly correlates with the fitness of the protein‐of‐interest. Within a couple of days, many cycles of error‐prone replication and in situ selection have occurred with minimal human intervention, like an *in vivo* PCR reaction. To date, the PACE system has mainly been used to optimise DNA‐binding proteins. To allow for optimisation of other enzymes, a prototype for a smart screening/selection system has been established by using an *E. coli* cell equipped with a specific signal transduction pathway that couples the enzyme‐based generation of a product to the growth/survival of the bacterial clone (Van Sint Fiet et al., 2006).

Another ground‐breaking development in laboratory evolution concerns the use of non‐biological compartments to maintain the genotype‐and‐phenotype link. Microtiter plates have frequently been used for this purpose. However, when high‐throughput analysis of large libraries is required, *in vitro* compartmentalisation (IVC) seems a better choice (Tawfik and Griffiths 1998). In IVC, single gene variants of a library are engulfed in artificial compartments such as water‐in‐oil droplets, or water‐in‐oil‐in‐water droplets. Recent progress in microfluidic technology has allowed for the production of highly monodisperse droplets (reviewed by Bouzetos et al., 2021). Gene expression inside these artificial compartment is catalysed by *in vitro* transcription and translation systems. Again, linking genotype and phenotype allows for enriching the genes encoding well‐performing enzyme variants. In case of general enzymes, this requires a covalent link of the enzyme's substrate and the enzyme‐encoding gene, which may be technically challenging; in case of DNA‐targeting enzymes this is straightforward: the nuclease gene and its target can easily be combined on a single synthetic DNA fragment.

The combination of evolution principles with the emerging technologies will be extremely powerful. Hence, efficiencies should be improved at all levels: generation of genetic libraries, compartmentalisation, and last but not least smart screening or selection approaches. Especially major recent developments of both microfluidics technology and of high‐throughput automated sorting methods of cells or droplets (FACS/FADS) hold promise for unprecedented possibilities in the (near) future to obtain proteins with desired optimal features (reviewed by Bouzetos et al., 2021). As Charles Darwin stated: ‘There is grandeur in this view of life, (…) from so simple a beginning endless forms most beautiful and wonderful have been and are being evolved’. This is not only true for biological creatures, but certainly also for their proteins. Hence, expectations for the future are high: from evolution to revolution!

## Conflict of interest

None declared.

## Funding information

No funding information provided.
